# Transition of microbial community structures after development of membrane fouling in membrane bioreactors (MBRs)

**DOI:** 10.1186/s13568-020-0959-2

**Published:** 2020-01-28

**Authors:** Yuya Sato, Yan-Jie Zhao, Tomoyuki Hori, Tomo Aoyagi, Tomohiro Inaba, Hidenobu Aizawa, Atsushi Ogata, Hiroshi Habe

**Affiliations:** Environmental Management Research Institute, National Institute of Advanced Industrial Science and Technology (AIST), 16-1 Onogawa, Tsukuba, Ibaraki 305-8569 Japan

**Keywords:** Membrane bioreactor, High-throughput sequencing, Microbial community, Membrane fouling, Activated sludge

## Abstract

Although membrane fouling is a major issue when operating membrane bioreactors (MBRs), information regarding MBR performance and the sludge microbiome after the development of fouling remains limited. For the present study, two MBRs were operated for approximately 1 month under conditions of membrane fouling to investigate the effects of highly stressed environments on the sludge microbiome. After the development of fouling, a *Collimonas*-related operational taxonomic unit (OTU) was highly dominant in both reactors (relative abundances were ⁓ 63%) and this predomination caused a precipitous decline in the diversity indices of the sludge microbiomes. Because the excessive predomination by limited numbers of OTUs can lead to reductions in the adaptability to environmental changes, monitoring microbial diversity may be a valuable indicator for maintaining the robustness of a sludge microbiome. While, the decrease in the abundance of the *Collimonas*-related OTU resulted in the predomination of distinct microorganisms in each of the reactors despite being operated under the same conditions; this finding indicates existence of strong pressure to perturb the microbiomes. Detailed analyses suggested that the availability of terminal electron acceptors and competitive interactions between microbes via the secretion of extracellular proteins appeared to differentiate the structures of the respective microbial communities. During the extracellular proteins were secreted in the sludge, considerable portion of microbes were dead and large amounts of biomolecules seemed to be released; resultantly facilitated the predomination of fermentative anaerobes in one reactor as they use organic substances but not inorganic terminal electron acceptors to generate ATP under anaerobic conditions.

## Introduction

Activated sludge has been used around the world for wastewater treatment for over 100 years and represents an important and familiar biotechnology in modern societies (Li et al. 2015; Sheik et al. 2014). While, activated sludge is a complex microbial community composed of more than thousand kinds of microorganisms including bacteria, archaea, protozoa, metazoa, and viruses (Albertsen et al. 2013; Inaba et al. 2018; Wuet al. 2019); this complexity has impeded our precise understanding of the characteristics of activated sludge (Griffin and Wells 2017). Previous studies have identified the constituent members of the sludge microbiome and revealed a close relationship between the structure of a microbial community and the treatment performance of activated sludge (Xia et al. 2018; Navarro et al. 2016; Sato et al. 2016b). However, a microbial population can be easily altered in response to environmental changes and, thus, it is challenging to operate an activated sludge bioreactor with a suitable microbiome over a long-term period in a stable manner (Vuono et al. 2015; Narihiro et al. 2019; Sato et al. 2019).

Membrane bioreactors (MBRs) combine the activated sludge process and membrane filtration and have become more popular in recent years because they offer several advantages over the conventional activated sludge process; i.e., high-quality effluent and a small footprint (Le-Clech 2010). However, a major drawback associated with MBRs is the occurrence of membrane fouling, which significantly reduces membrane performance and increases maintenance and operation costs. Although various biological and physicochemical indicators that can forecast membrane fouling have been evaluated (Inaba et al. 2017; Wang et al. 2009), almost all studies investigating this issue were performed prior to the development of membrane fouling. Therefore, information regarding the sludge microbiome after membrane fouling remains limited despite the fact that these unfavorable operational conditions are conceivable in practice. It can be predicted that the activated sludge microorganisms would be highly stressed during the operation of an MBR with a fouled membrane because undegraded organic compounds in wastewater can remain inside the sludge for long periods due to a reduced effluent rate and increases in the organic loading rate within the system (Inaba et al. 2018). Together, these factors may result in strong pressure that can perturb microbial populations in the activated sludge (Sato et al. 2016b, c; Vuono et al. 2015).

The present study operated two pilot-scale MBRs with excessively high flow rates to facilitate the development of membrane fouling. Subsequently, the MBRs were operated with fouled membranes for approximately 1 month to investigate the effects of a highly stressed environment on the sludge microbiome. In this report, the obtained microbiome data are comparatively discussed with physicochemical data regarding MBR performance to further assess previously unknown indicators of decline in MBR performance.

## Materials and methods

### Operational conditions of the pilot-scale MBRs

For the present study, two replicate bioreactors with 230-liter volumes were operated under the same conditions in which a membrane module with a 0.24-m^2^ flat polyacrylonitrile membrane (0.07 µm pore size, M-fine, Awa Paper Mfg. Co.; Tokushima, Japan) was submerged in the reaction tank (Sato et al. 2015). The flow rates of both the input wastewater and the output membrane-filtered treated water were initially set to 576 l day^−1^, which resulted in a hydraulic retention time of 0.4 days. The return sludge from the third to the first compartment had a flow rate of 115 l day^−1^, the membrane filtration was performed with a cycle of permeate extraction for 9 min and a pause for 1 min, and, to mix the activated sludge and control dissolved oxygen (DO) levels, air was provided through an air diffuser set at the bottom of each compartment at a flow rate of 12.5–30.0l min^−1^. The activated sludge in the present study was obtained from a municipal wastewater treatment plant (Kinu Aqua-station; Ibaraki, Japan) and the initial mixed liquor suspended solids (MLSS) concentration was approximately 6000 mg l^−1^; no sludge was withdrawn from the reactor during the operation.

The concentration of the inlet synthetic wastewater was set at 450 mg chemical oxygen demand (COD) l^−1^ (1130 total organic carbon [TOC] mg l^−1^) by diluting the concentrated synthetic wastewater solution with tap water, and contained CH_3_COONa (2.65 g l^−1^), NH_4_Cl (0.376 g l^−1^), KH_2_PO_4_ (0.109 g l^−1^), and peptone (0.706 g l^−1^) as well as the trace elements FeCl_3_·6H_2_O (0.782 mg l^−1^), CaCl_2_ (1.56 mg l^−1^), MgSO_4_ (1.56 mg l^−1^), KCl (1.56 mg l^−1^), and NaCl (1.56 mg l^−1^). The organic loading rate was calculated to 1125 mg COD l^−1^ day^−1^.

To induce membrane fouling, the reactors were operated at a flow rate of 100 l m^−2^ h^−1^, which is excessively higher than the flux value of approximately 20 l m^−2^ h^−1^ that was previously reported to be necessary for stable operation without membrane fouling (Navaratna and Jegatheesan 2011). After fouling had developed, the two reactors continued to be operated using the fouled membrane for approximately 1 month; during this period, the concentrated synthetic wastewater was continuously fed into MBRs to maintain the same organic loading rate. The MLSS, temperature, DO, pH, and transmembrane pressure (TMP) of the membrane module were monitored throughout the experimental period. Membrane cleaning with 0.1% NaOCl was performed when required to continue MBR operation for long by reducing the foulants. Sampling of the activated sludge and the effluent (i.e., treated water) were performed at daily intervals. The obtained activated sludge samples were centrifuged (15,300×*g*, 15 min, and 4 °C) and the resulting supernatants and pellets were stored at −20 °C separately until further analysis.

## 16S rRNA genetic analysis

The microbial cell pellets collected daily from the activated sludge samples were used for the microbial analyses. Genomic DNA was extracted using a direct lysis protocol that included chemical lysis, bead-beating, phenol–chloroform extraction, and ethanol precipitation (Aoyagi et al. 2015). The universal primer sets of 515F and 806R were used to amplify the V4 region of 16S rRNA genes with a high-fidelity DNA polymerase (Q5, NEB; Ipswich, MA, USA); both primers were modified to contain an Illumina adapter region and the reverse primer contained a 12-bp barcode for multiplex sequencing (Caporaso et al. 2012). The polymerase chain reaction (PCR) conditions were the same as previously described (Sato et al. 2016c), except that 30–35 cycles were performed. The PCR product was first purified with an AMPure XP kit (Beckman Coulter; Brea, CA, USA) and the target DNA fraction was isolated from the incised gel and extracted using the Wizard SV Gel and PCR Clean-Up System (Promega; Madison, WI, USA). The concentration of the purified DNA was spectrophotometrically measured using a Quant-iT PicoGreen dsDNA reagent and kit (Life Technologies; Carlsbad, CA, USA) and the paired-end sequencing was conducted using a Miseq System (Illumina; San Diego, CA, USA) with a 500-cycle MiSeq reagent kit v2.

The PhiX sequences in the Illumina sequence libraries were removed using a homology search against the Greengenes database (McDonald et al. 2012). Subsequently, the obtained paired-end sequences were assembled and only high quality joined sequences (Phred value score [Q] ≥ 30) were collected using QIIME software version 1.7.0 (Caporaso et al. 2010). Next, these sequences were aligned by the mothur program version 1.31.2 (Schloss et al. 2009), which can also detect and exclude chimeric sequences from the library. QIIME software was used to phylogenetically analyze the sequences in each library as well as the α-diversity indices (i.e., Chao1, Shannon, and Simpson reciprocal) and weighted UniFrac distances for the principal coordinates analysis (PCoA) analysis (Lozupone et al. 2011). The species related to the predominant operational taxonomic units (OTUs) were further determined based on a BLAST search of the NCBI database (http://blast.ncbi.nlm.nih.gov/Blast.cgi).

### Sodium dodecyl sulfate polyacrylamide gel electrophoresis analyses

The extracellular proteins in the supernatants of the activated sludge were analyzed using sodium dodecyl sulfate polyacrylamide gel electrophoresis (SDS-PAGE). For these analyses, 20 µg samples of the supernatants were denatured under reducing conditions by boiling them at 95 °C for 5 min in the sample buffer (50 mM Tris, pH 6.8, 1% SDS, 2% β-mercaptoethanol, and 0.01% bromophenol blue) and then fractionating the samples in a 13% polyacrylamide gel via electrophoresis. The proteins in the gel were visualized using fluorescent staining with Orile (Bio-Rad Laboratories; Hercules, CA, USA). The protein concentrations in the supernatants were determined using Quick Start Bradford Protein Assay kits with bovine serum albumin as the standard (Bio-Rad Laboratories) and a microplate reader (SH-9000, Corona Electric, Ibaraki, Japan) (Sato et al. 2016a).

## Results

### Physicochemical analyses of the pilot-scale MBRs

**Fig. 1:**
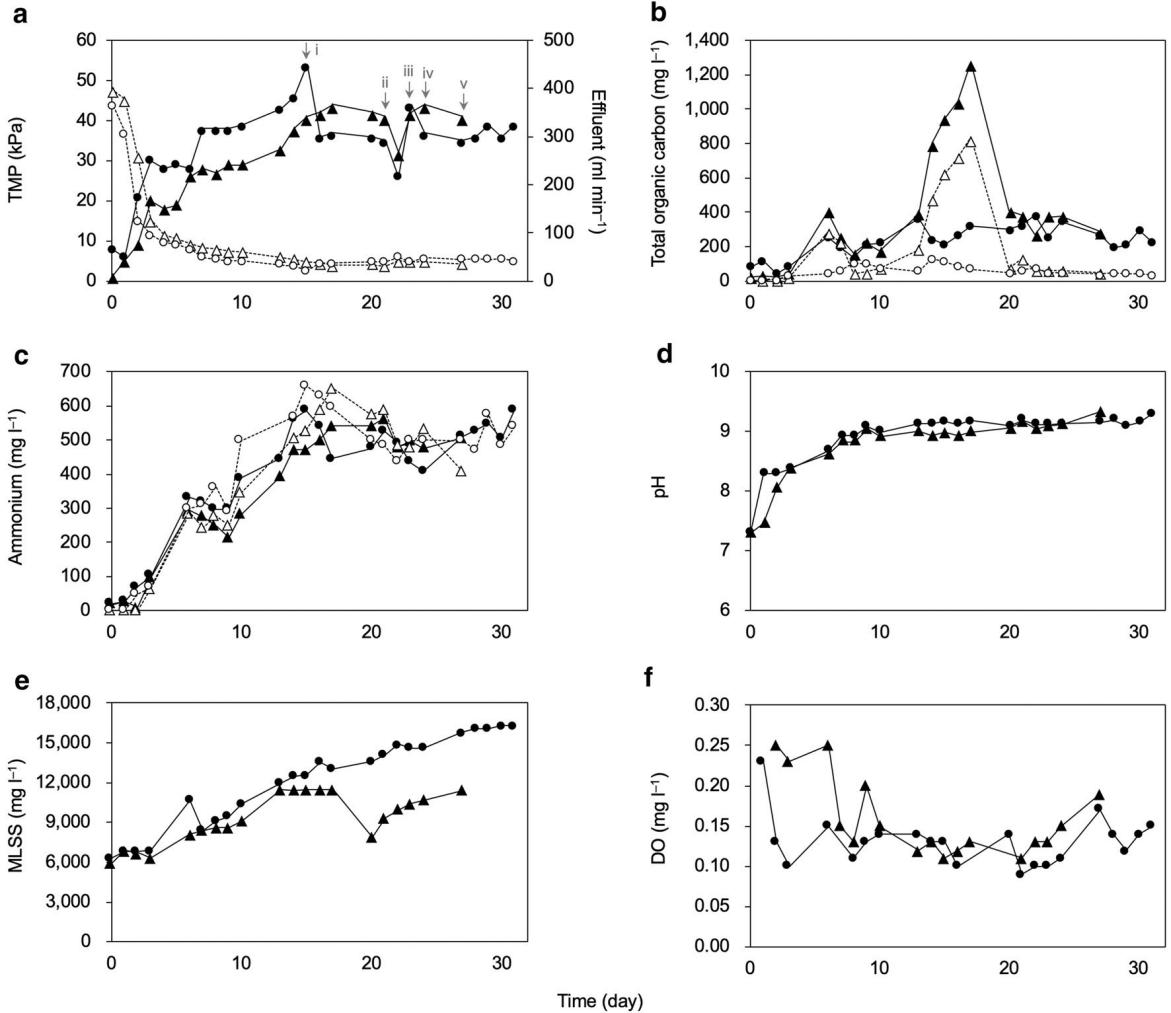
Physicochemical parameters in the membrane bioreactors. **a** Transmembrane pressure (TMP) and effluent flow rate; **b** total organic carbon (TOC) concentration; **c** ammonium concentration; **d** pH; **e** mixed liquor suspended solids (MLSS) concentration; **f** dissolved oxygen concentration. Circles and triangles denote the data for Reactors 1 and 2, respectively, and closed and open symbols denote the data detected in the sludge and effluent, respectively. The arrows in panel A indicate the day of membrane cleaning with 0.1% NaOCl; (i) Cleaning was performed on Day 15 in Reactor 1 and on Days 21, 23, and 24 (ii, iii and iv) in both reactors

The operations of two 230-liter volume pilot-scale MBRs, which were designated as Reactors 1 and 2, were started with an effluent flow rate of approximately 400 ml min^−1^; this rate was five times higher than the flow rate used in previous studies conducted by our research group (Fig. 1a) (Sato et al. 2016b, c). The TMP, which indicates the requisite pressure level for membrane filtration and is a widely used indicator of membrane fouling (Le-Clech 2010; Navaratna and Jegatheesan 2011), increased to 20–30 kPa in the first 4 days. Concomitant with the increase in TMP, the effluent flow rate drastically decreased to below 100 ml min^−1^. Taken together, these results indicate that membrane fouling was successfully developed by the excessively high flow rate conditions. Subsequently, the two MBRs continued to be operated for approximately one month (up to Days 31 and 27 for Reactors 1 and 2, respectively) with membrane cleaning performed on Days 15, 21, 23, and 24; recovery of the TMP and effluent flow rates were limited (Fig. 1a, i–iv).

The TOC concentrations in the two MBRs were similar, except for a spike in Reactor 2 on Days 13–17 (Fig. 1b). The average TOC concentrations on Days 0–3 were 82 and 29 mg l^−1^ in the activated sludge of Reactors 1 and 2, respectively, while those after Day 3 were 262 and 486 mg l^−1^, respectively, which implies that the sludge microorganisms were under high organic loading conditions following membrane fouling. By contrast, the average TOC concentrations in the effluent were several times lower than those in the sludge (61 and 211 mg l^−1^ in Reactors 1 and 2, respectively). Although fouled, it is possible that the membrane reduced the outflow of dissolved organic matter from the MBRs. The ammonium concentrations in the MBRs drastically increased after Day 3 and were then maintained at high levels throughout the operation; the average values were 461 and 434 mg l^−1^ in Reactors 1 and 2, respectively (Fig. 1c). Unlike the TOC values, the ammonium concentrations in the effluent were as high as those in the sludge, which indicates that the ammonium molecules passed through the fouled membrane. A similar increasing trend was observed in pH values, possibly due to the increase in ammonium concentrations (Fig. 1d) (Sato et al. 2016d).

The MLSS concentrations increased two-fold from Day 0 to 13 (approximately 6000 to 12,000 mg l^−1^) in both reactors (Fig. 1e). However, the MLSS concentration in Reactor 1 continued to increase throughout operation whereas the concentration in Reactor 2 decreased from Day 13 to 20. The DO concentrations in the two reactors were maintained at levels lower than 0.3 mg l^−1^ (Fig. 1f).

### Diversity indices of the sludge microbial communities

**Fig. 2:**
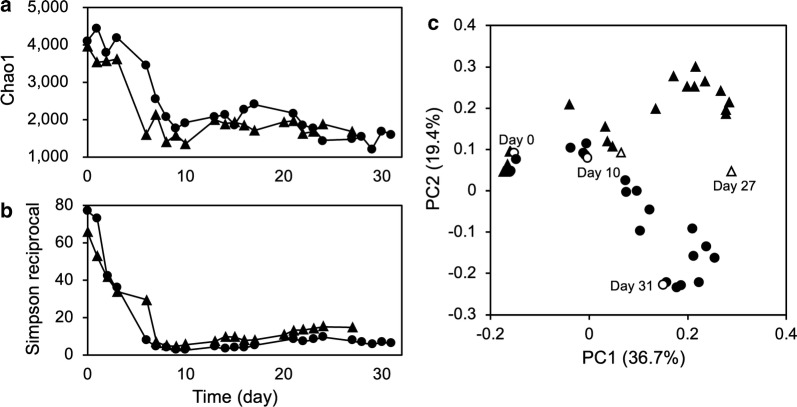
Alpha-diversity indices and the PCoA plot based on the sequence data. **a** Chao1; **b** simpson reciprocal. Each index was calculated based on an equal amount of sequences (19,218 and 14,598 for Reactors 1 and 2, respectively) that were sub-sampled from the original libraries 10 times. For each index, a higher value represents a more diverse microbial community. **c** PCoA scatter plot of 16S rRNA sequences based on the weighted UniFrac distances. Circles and triangles denote Reactors 1 and 2, respectively

The α-diversity indices (i.e., Chao 1 and Simpson reciprocal) of the sludge microbial communities in Reactors 1 and 2 showed essentially similar trends (Fig. 2a, b). Both the Chao1 and Simpson reciprocal indices, which primarily denote the richness and evenness of microbial species in the community, had drastically decreased at Day 9 and then maintained low levels during operation. These results suggest that large numbers of microbial species were washed out during operation under the high organic loading conditions and that only small numbers of microorganisms dominated the sludge microbial communities. The similarity of the microbial community structures in the two reactors was compared with a PCoA scatter plot (Fig. 2c). On Day 4, the microbial communities in the two reactors were stable and similar to each other and, even after the development of membrane fouling, the two microbial communities showed similar structural shifts on Days 6–10. However, after Day 10, the community structures appeared to largely differ from one another despite the same operational conditions.

### Compositional changes of the microbial communities at the class and genus levels

**Fig. 3:**
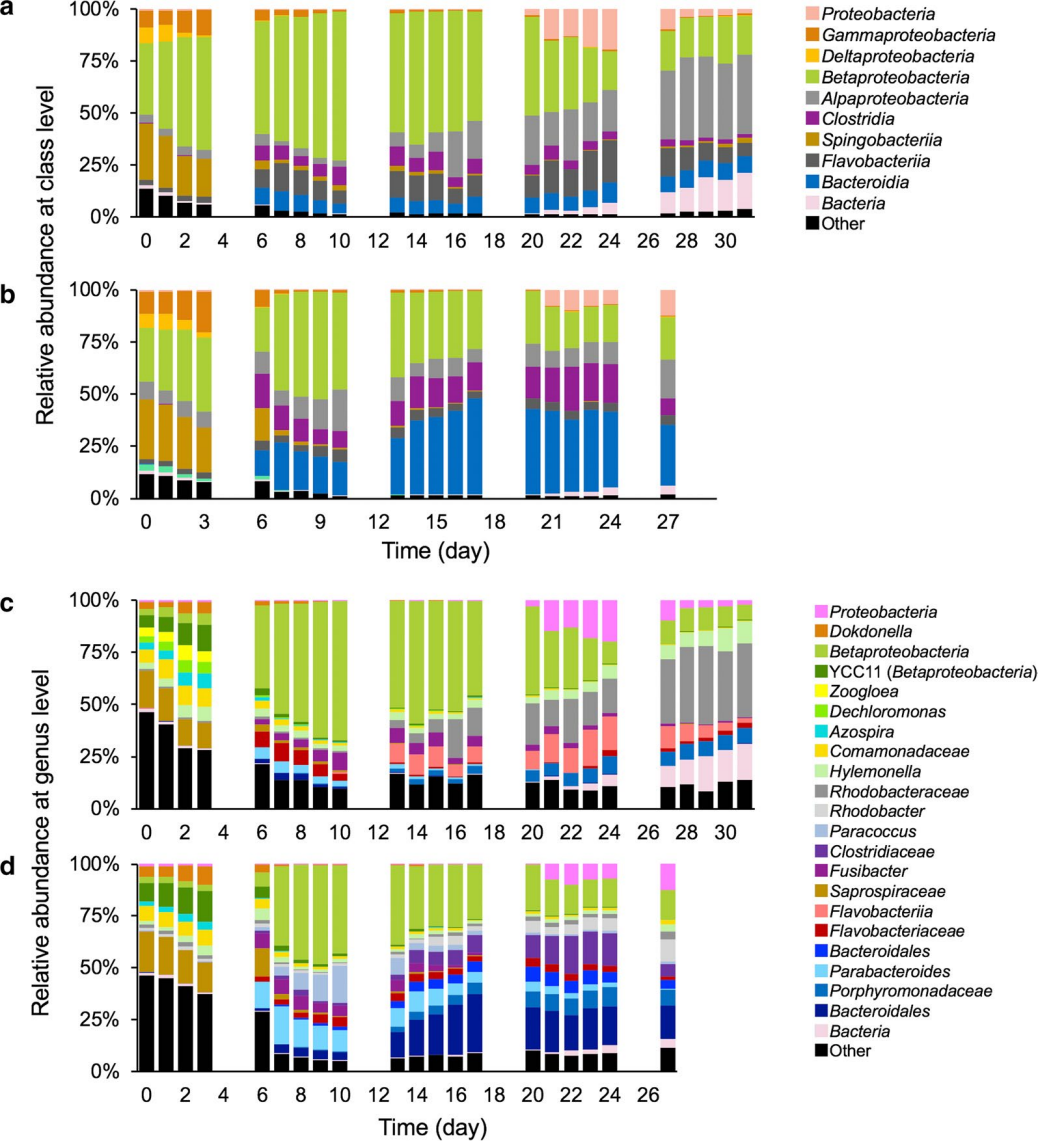
Structural changes in the microbial communities at the class and genus levels throughout the operation. **a**, **b** Analysis at the class level in Reactors 1 and 2, respectively. **c**, **d** Analysis at the genus level in Reactors 1 and 2, respectively. The phylogenetic groups (class or genus) are indicated by color and their taxonomies are shown to the right of the graphs. Phylogenetic categories were assigned using QIIME software

The compositional changes of the microbial communities that occurred throughout operation of the MBRs were analyzed using QIIME software (Fig. 3a–d). On Day 4, the microbial community structures in the two reactors were similar and relatively stable and, even after the development of membrane fouling (i.e., at Days 6–10), the two microbial communities showed similar structural changes in which a genus belonging to *Betaproteobacteria* highly dominated the sludge microbial community (Fig. 3a–d). However, the two sludge communities exhibited distinct structural shifts after Day 10; the series of structural changes corresponded well with the results of the PCoA plot (Fig. 2c). Concomitant with the decrease in the abundance of *Betaproteobacteria*, there was a predominance of *Alphaproteobacteria* and an unidentified class in the microbial community in Reactor 1 while *Clostridia* and *Bacteroidia* predominated the microbial community in Reactor 2 (Fig. 3a, b).

### Phylogenetic analysis of the dominant OTUs

**Fig. 4:**
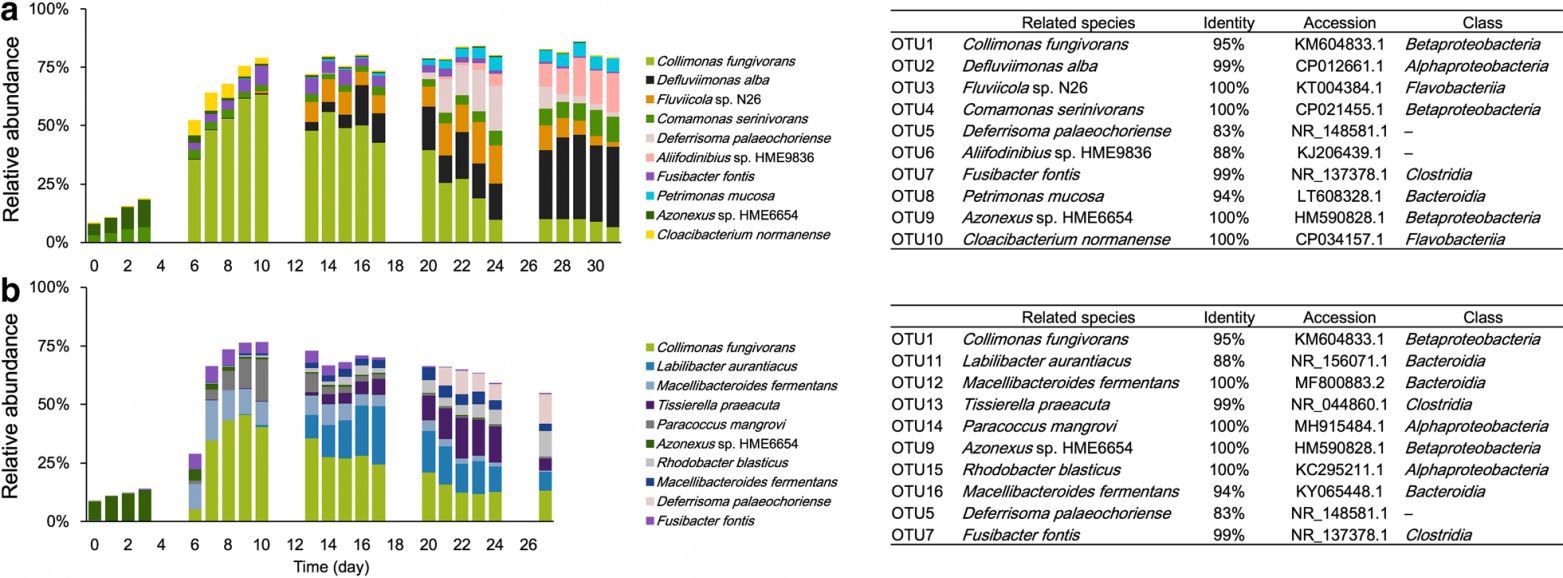
Transition of the 10 most dominant operational taxonomic units (OTUs) throughout the operation. Microbial community structures and phylogenetic information in Reactors 1 (**a**) and 2 (**b**). The phylogenetic information of each OTU is shown in the table on the right. The related species of the OTUs were assigned using a BLAST analysis (https://blast.ncbi.nlm.nih.gov/Blast.cgi) and the phylogenetic categories at a class level were assigned using QIIME software

The detailed phylogenetic information and relative abundances of the 10 most common OTUs in the two reactors are summarized in Fig. 4. The first structural shifts in the microbial communities in the two reactors were similar at the OTU level (Days 6–10) such that OTU1 highly predominated the sludge microbiome; the relative abundances in Reactors 1 and 2 were ~ 63% at Day 10 and ~ 46% at Day 9, respectively. The closest relative of this OTU was *Collimonas fungivorans*, which is capable of feeding on living fungal hyphae by secreting lytic enzymes (e.g., chitinase, peptidase, and phospholipase) and/or antimicrobial secondary metabolites (Song et al. 2015). Although OTU1 did not appear to actually be *Collimonas fungivorans* because its sequence identity was 95%, it might have possessed a similar ability to eliminate competing microorganisms. Regardless, the predominance of *Collimonas fungivorans* appeared to suppress the predomination of other microorganisms and resulted in precipitous declines in the evenness of the microbial communities in the two reactors.

After Day 10, phylogenetically distinct microorganisms predominated the sludge microbiomes of the respective reactors; i.e., OTUs 2, 3, 4, 5, 6, and 8 in Reactor 1 and OTUs 5, 11, 13, 15, and 16 in Reactor 2. The species related to OTUs 2, 3, 4, 5, 6, and 8 were *Defluviimonas alba* (with a sequence identity of 99%), *Fluviicola* sp. N26 (100%), *Comamonas serinivorans* (100%), *Deferrisoma palaeochoriense* (83%), *Aliifodinibius* sp. HME9836 (88%), and *Petrimonas mucosa* (94%), respectively. *Defluviimonas alba* is an aerobe capable of using oxygen as the terminal electron acceptor whereas most *Defluviimonas* species are facultative anaerobes that are also capable of nitrate respiration (Zhao et al. 2016). *Fluviicola* spp. are basically aerobic but it has been reported that one species is weakly positive for nitrate reduction (Yang et al. 2014). *Comamonas serinivorans* is a facultative anaerobe capable of nitrate reduction (Zhu et al. 2014). *Petrimonas mucosa* is a facultative anaerobe that uses fermentation under anaerobic conditions (Hahnke et al. 2016). OTUs 5 and 6 seemed phylogenetically distinct from previously described microorganisms (sequence homologies with most of the related species were < 90%).

The species related to OTUs 5, 11, 13, 15, and 16 (the dominant OTUs in Reactor 2) were *Deferrisoma palaeochoriense* (with a sequence identity of 83%), *Labilibacter aurantiacus* (88%), *Tissierella praeacuta* (99%), *Rhodobacter blasticus* (100%), and *Macellibacteroides fermentans* (94%), respectively. *Tissierella praeacuta* is an obligate anaerobe but its usage of terminal electron acceptors remains unclear (Collins and Shah 1986), while, some species belonging to this genus have been reported to utilize proteinaceous substrates for their growth in anaerobic digestion processes (Nolla-Ardevol et al. 2015). *Rhodobacter blasticus* is an aerobic photosynthetic bacterium while *Macellibacteroides fermentans* is an obligate anaerobe that uses anaerobic fermentation (Brosché et al. 1998; Jabari et al. 2012).

### Extracellular proteins in the activated sludge

**Fig. 5:**
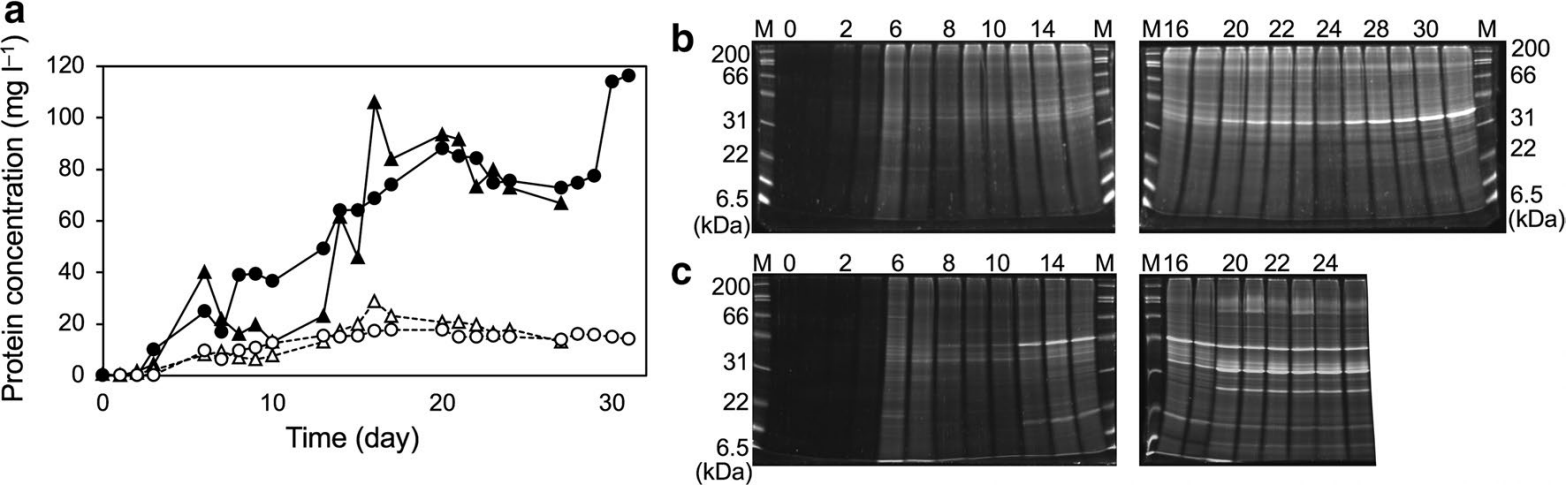
Extracellular protein concentration and size distribution profiles in the activated sludge supernatants. **a** Protein concentrations in the sludge supernatants (closed symbols) and the effluent (open symbols). Circles and triangles denote Reactors 1 and 2, respectively. **b, c** SDS-PAGE analyses of the supernatants of the activated sludge in Reactors 1 and 2, respectively. Labels on the top denote operational days or size maker (M). Labels on the left denote size of the marker proteins

The concentration and size distribution profiles of the extracellular proteins in the supernatant of the activated sludge were analyzed using spectrophotometric measurements and SDS-PAGE, respectively (Fig. 5). The extracellular protein concentrations were higher in the activated sludge supernatants than in the effluent in both reactors, which indicates that a majority of the extracellular proteins did not pass through the membrane unit and remained in the activated sludge (Fig. 5a). Notably, the protein concentrations sharply increased on Days 13–17 in Reactor 2 (Fig. 5a), which corresponded to the trend of TOC levels in the same reactor (Fig. 1b). Furthermore, sharp protein bands appeared within the same time period with SDS-PAGE analysis of Reactor 2, which indicates that the proteins were secreted out of the microbial cells rather than leaked from damaged cells (Fig. 5b, c).

## Discussion

The two MBRs were operated with the fouled membrane for around 1 month. Even though the membrane fouling had been developed, TOC removal ratio was relatively high during the operation (94.6% and 81.3% in Reactor 1 and 2, respectively, on average), whereas the nitrogen removal rate was quite low (Fig. 1b, c). Under the high organic loading conditions after the development of membrane fouling, DO level in the sludge was low (Fig. 1f), even though the activated sludge was continuously aerated throughout the operation. This indicates that there was a high demand for electron acceptors in the activated sludge under high organic loading conditions (Navarro et al. 2016; Sato et al. 2019), while electron donors (i.e., organic compounds in synthetic wastewater, such as peptone) were abundantly available.

The PCoA analysis suggests that the development of membrane fouling initially induced similar structural shifts in the two microbial communities (Fig. 2c; Days 6–10) but then the sludge microbial communities underwent further structural changes in divergent direction due to respective environmental factors. This finding indicates the presence of strong pressure to perturb the microbiomes under high organic loading conditions (Sato et al. 2019). The phylogenetic analyses demonstrated that class *Betaproteobacteria* (mainly OTU1) once highly dominated in the both reactors (Fig. 3a, b); this predominance is corresponded to decrease in the diversity indices (Fig. 2a, b) and shift in PCoA plot on Days 6–10 (Fig. 2c). However, after Day 10, Reactor 1 was predominated by *Alphaproteobacteria* and unidentified class, whereas *Clostridia* and *Bacteroidia* predominated in Reactor 2 (Fig. 3a, b). Notably, *Alphaproteobacteria* are mainly composed of aerobes and facultative anaerobes whereas *Clostridia* and *Bacteroidia* are mainly composed of obligate anaerobes.

The 10 most abundant OTUs in Reactor 1 were aerobes or facultative anaerobes, rather than obligate anaerobes. By contrast, the dominant OTUs in Reactor 2 were strictly aerobes or obligate anaerobes that use anaerobic fermentation. These results suggest that oxygen was an important and a commonly used terminal electron acceptor in both reactors, possibly because aerobic respiration is the most efficient strategy for generating adenosine triphosphate (ATP). The next most efficient ATP generating strategy involves nitrate reduction (Orcutt et al. 2011). The nitrate concentrations in the two reactors were below detection limits after Day 2 (Additional file 1: Fig. S1A), indicating the importance of nitrate as the terminal electron acceptor under low oxygen conditions in both reactors while the denitrifiers did not predominate in Reactor 2 (Fig. 4b). By contrast, there were obvious differences in sulfate concentrations between the two reactors (Additional file 1: Fig. S1B) such that the sulfate concentration in Reactor 1 increased after Day 20 whereas it was below the detection limit in Reactor 2, which implies that sulfate was preferably required for the microbiome in Reactor 2 but not in Reactor 1. Even though sulfate-reducing bacteria were not found among the dominant OTUs, other low-abundance microorganisms in Reactor 2 might have used sulfate as an electron acceptor. Besides, the abundances of nitrifiers, i.e., ammonia oxidizing bacteria and nitrite oxidizing bacteria, at Day 1–4 were apparently high in Reactor 1 than in Reactor 2 (Additional file 1: Fig. S2). Low level of nitrate production from ammonia might allow the microbiome in Reactor 2 to preferably utilize the other terminal electron acceptors, such as sulfate, in the anaerobic environments possibly existing locally in the activated sludge.

To further investigate the factors caused the difference in microbial community structures, a particular focus was placed on extracellular proteins because on Days 13–17 during which the structural shifts in the two microbial communities appeared to begin, the extracellular protein concentration sharply increased while the MLSS value decreased in only Reactor 2 (Figs. 1e and 4a). Because some microorganisms express extracellular lytic enzymes to compete for survival in complex microbial communities (Lakshmi et al. 2014), the extracellular proteins observed in the SDS-PAGE analyses might have had such functions. The fact that the MLSS concentration in Reactor 2 decreased in the same time period may support this hypothesis. To identify the proteins, the supernatants of the activated sludge were fractionated using fast protein liquid chromatography with an ion exchange column. Although one of the five proteins detected in the SDS-PAGE analyses was successfully purified, neither N-terminal amino acid sequencing using a protein sequencer nor an ion search using nano-liquid chromatography tandem mass spectrometry could identify any proteins (data not shown). To figure out the effect of the extracellular proteins on the microbial community in Reactor 2, the OTUs whose relative abundance highly increased on Days 10–20, in which the protein concentration increased and the MLSS value decreased, were summarized in Additional file 1: Table S1. Notably, 11 of the 20 OTUs were affiliated within *Bacteroidia*; all of these species are fermentative anaerobes (including facultative and obligately ones) that can utilize organic substances to generate ATP under anaerobic conditions (Grabowski et al. 2005; Jabari et al. 2012). The decrease in MLSS value and the increase in TOC and protein concentrations on Days 10–20 suggested that, during this time period, abundant biomolecules were discharged from the damaged microbial cells. Such environment seemed advantageous for anaerobic fermentative microorganisms, possibly allowing the predominance of the 11 *Bacteroidia* OTUs in Reactor 2.

In conclusion, the present study observed the dynamic transition of sludge microbial communities under high organic loading conditions following the induction of membrane fouling, which is an issue that has yet to be fully investigated. One disadvantage associated with wastewater treatment that is caused by extreme conditions may be the excessive predomination of limited numbers of OTUs because this type of predominance can reduce the diversity of a sludge microbiome and subsequently decrease adaptability to environmental changes (Loreau et al. 2003). Therefore, monitoring microbial diversity during the operation of activated sludge bioreactors might be a valuable indicator for maintaining the robustness of a sludge microbiome. On the other hand, the microbial communities in the two reactors initially showed similar structural shifts but underwent additional changes in different directions even though the two reactors were operated under the same conditions. The microbial community structures seemed to be shaped basically by the availability of terminal electron acceptors. Further, spike in concentration of organic compound appeared to facilitate predominance of anaerobic fermentative microorganisms and changed the community structure of Reactor 2.

## Supplementary information

**Additional file 1: Fig. S1. a** Nitrate and **b** sulfate concentrations in the effluents of Reactors 1 and 2; the values are means of two measurements. Bars indicate variations and circles and triangles denote the values for Reactors 1 and 2, respectively. **Fig. S2.** Relative abundances of OTUs assigned to nitrifiers in Reactors 1 (**a**) and 2 (**b**). Phylogenetic classification of the OTUs was performed using BLAST analysis (https://blast.ncbi.nlm.nih.gov/Blast.cgi). **Table S1.** OTUs that highly increased from day 10 to 20 in Reactor 2.

## Data Availability

The raw sequence data obtained in the present study have been deposited in the DNA Data Bank of Japan (DDBJ) Sequence Read Archive under accession code DRA008692.
