# Supplementary material for: Transition of microbial community structures after development of membrane fouling in membrane bioreactors (MBRs)
Source: AMB Express. 2020 Jan 28;10:18. doi: 10.1186/s13568-020-0959-2 (PMC6987300; doi:10.1186/s13568-020-0959-2)
Supplement: Supplementary file 1 — Additional file 1: Fig. S1. a Nitrate and b sulfate concentrations in the effluents of Reactors 1 and 2; the values are means of two measurements. Bars indicate variations and circles and triangles denote the values for Reactors 1 and 2, respectively. Fig. S2. Relative abundances of OTUs assigned to nitrifiers in Reactors 1 (a) and 2 (b). Phylogenetic classification of the OTUs was performed using BLAST analysis (https://blast.ncbi.nlm.nih.gov/Blast.cgi). Table S1. OTUs that highly increased from day 10 to 20 in Reactor 2. [file 13568_2020_959_MOESM1_ESM.pdf]

Additional file 1

AMB Express

Title

Transition of microbial community structures after development of membrane fouling in membrane bioreactors (MBRs)

Authors

Yuya Sato[†,*], Zhao Yan-Jie[†], Tomoyuki Hori, Tomo Aoyagi, Tomohiro Inaba, Hidenobu Aizawa, Atsushi Ogata and Hiroshi Habe

[†]The authors equally contributed to this work.

*Corresponding author

Corresponding authors

Yuya Sato

Environmental Management Research Institute, National Institute of Advanced Industrial Science and Technology (AIST), 16-1 Onogawa, Tsukuba, Ibaraki 305-8569, Japan

E-mail: yuya-satou@aist.go.jp

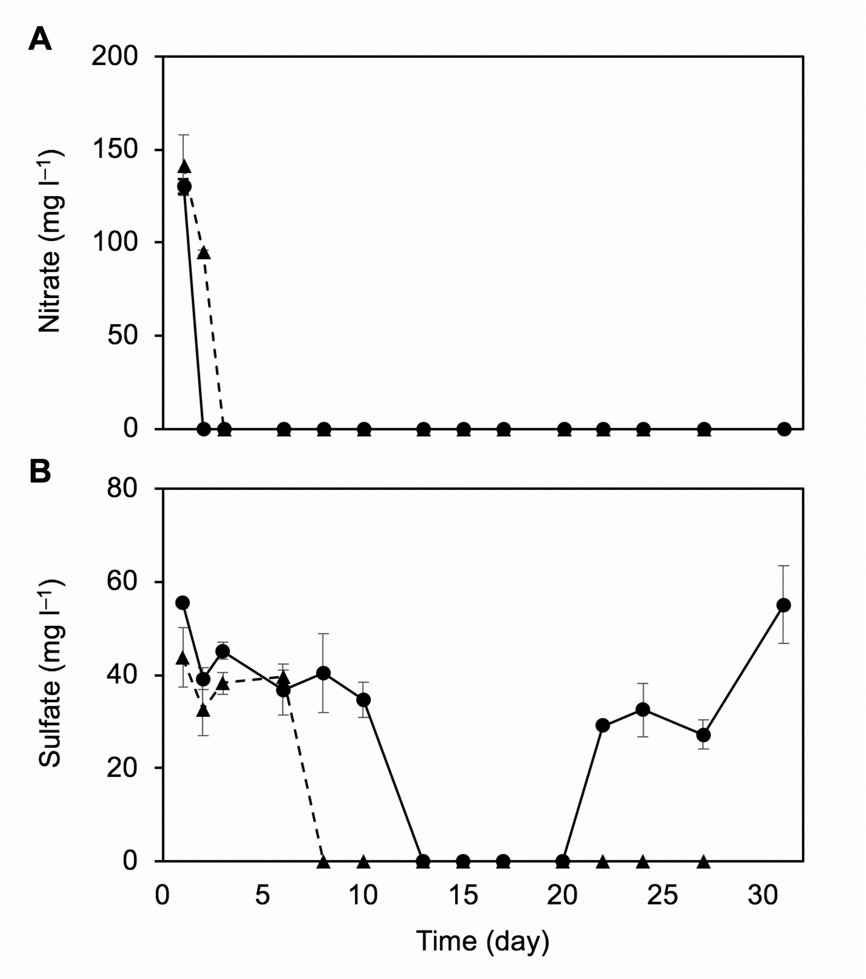

Supplemental figure S1. (**A**) Nitrate and (**B**) sulfate concentrations in the effluents of Reactors 1 and 2; the values are means of two measurements. Bars indicate variations and circles and triangles denote the values for Reactors 1 and 2, respectively.

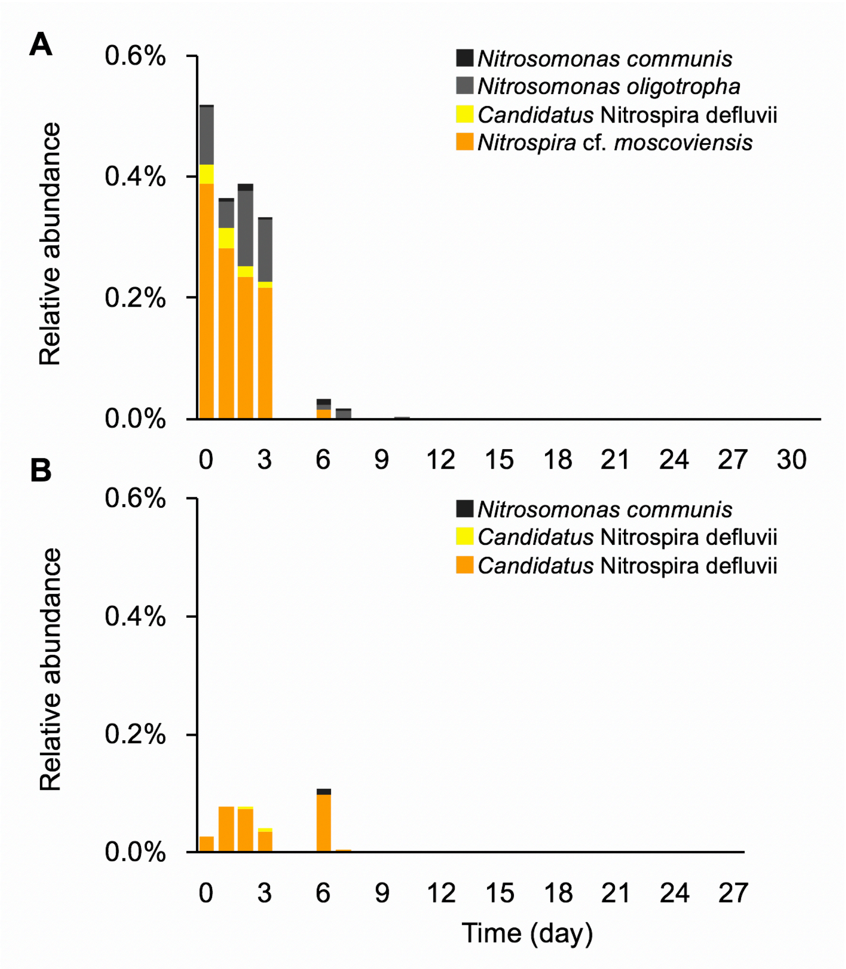

Supplemental figure S2. Relative abundances of OTUs assigned to nitrifiers in Reactors 1 **(A)** and 2 **(B)**. Phylogenetic classification of the OTUs was performed using BLAST analysis (https://blast.ncbi.nlm.nih.gov/Blast.cgi).

Supplemental table S1. OTUs that highly increased from day 10 to 20 in Reactor 2.

	Increasing ratio	Related microorganisms	Identity	Accession	Class[a]
OTU13	41.2	*Tissierella praeacuta*	99%	NR_044860.1	*Clostridia*
OTU17	34.3	*Sedimentibacter acidaminivorans*	95%	NR_148817.1	*Clostridia*
OTU18	30.5	*Petrimonas sulfuriphila*	97%	LT558828.1	*Bacteroidia*
OTU19	28.3	*Macellibacteroides fermentans strain*	94%	MF800883.2	*Bacteroidia*
OTU20	24.9	*Alcaligenes faecalis*	100%	CP036294.1	*Betaproteobacteria*
OTU21	22.3	*Paracoccus contaminans*	99%	CP020612.1	*Alphaproteobacteria*
OTU22	21.6	*Petrimonas sulfuriphila*	98%	LT558828.1	*Bacteroidia*
OTU11	16.7	*Labilibacter aurantiacus*	88%	NR_156071.1	*Bacteroidia*
OTU23	15.8	*Petrimonas mucosa*	94%	LT608328.1	*Bacteroidia*
OTU24	15.0	*Ammoniphilus oxalaticus*	87%	MF193690.1	*Clostridia*
OTU25	12.9	*Paracoccus huijuniae*	98%	KJ001153.1	*Alphaproteobacteria*
OTU26	12.7	*Petrimonas sulfuriphila*	96%	KT183420.1	*Bacteroidia*
OTU27	12.4	*Petrimonas sulfuriphila*	96%	LT558828.1	*Bacteroidia*
OTU28	10.3	*Imtechella halotolerans*	94%	NR_117181.2	*Flavobacteriia*
OTU29	10.3	*Methylocystis* sp. strain B2-1	97%	U81595.1	*Alphaproteobacteria*
OTU30	9.0	*Dysgonomonas gadei*	95%	NR_113134.1	*Bacteroidia*
OTU31	8.6	*Macellibacteroides* sp. strain T1.2MG-59	96%	MF361873.1	*Bacteroidia*
OTU33	8.2	*Macellibacteroides fermentans*	92%	MF800883.2	*Bacteroidia*
OTU32	8.2	*Stenotrophobacter terrae*	97%	NR_146023.1	*Chloracidobacteria*
OTU34	7.7	*Macellibacteroides fermentans*	90%	MF800883.2	*Bacteroidia*

[a]Phylogenetic classification was performed using QIIME softwatre with Greengenes database.
